# Seasonal Influenza Vaccination Uptake and Intentions Among Nursing Students in Hong Kong

**DOI:** 10.3390/vaccines13121252

**Published:** 2025-12-17

**Authors:** Maria Shuk Yu Hung, Grace Sun King Wan, Wai Hon Chua, Ching Man Cheng

**Affiliations:** S.K. Yee School of Health Sciences, Saint Francis University, Hong Kong, China

**Keywords:** seasonal influenza, vaccination, nursing students, Hong Kong

## Abstract

**Background/Objectives:** Seasonal influenza is a global public health issue, and influenza vaccination is the most effective preventive measure. Nursing students are at a higher risk of contracting it due to clinical exposure. However, vaccination uptake among nursing students remains low. This study examines seasonal influenza vaccination uptake rates and the predictors of nursing students’ willingness to receive this vaccine. **Methods:** A cross-sectional online study was conducted using a well-validated questionnaire in order to collect data from a convenience sample of nursing students in Hong Kong from early November to early December 2024. **Results:** Out of the 461 valid responses received, 67.5% were from females, with a mean age of 20.53 (SD = 2.16) years. Vaccination history was diverse: 34.3% reported that they underwent influenza vaccination in the most recent season, 49.7% reported that they were vaccinated one to two times over the preceding five years, and only 5% reported that they undergo vaccination annually. A logistic regression model showed that the respondents who had been vaccinated against influenza in the most recent years (OR = 2.881, 95% CI: 1.773–4.680) had been vaccinated against influenza 1–2 times (OR = 3.239, 95% CI: 1.750–5.993), had been vaccinated 3–4 times (OR = 3.984, 95% CI: 1.773–8.957), had been vaccinated every year (OR = 10.353, 95% CI: 3.025–35.436) in the past five years, and had a higher score of perceived susceptibility (OR = 2.244, 95% CI: 1.302–3.867) were more likely to intend to be vaccinated with an influenza vaccine in the coming year. **Conclusions:** Annual seasonal influenza vaccination rates among nursing students remain low, though they have improved. We recommend that the Hong Kong government, healthcare organizations, and universities develop effective strategies for promoting annual seasonal influenza vaccination to safeguard both patients’ health and the health of future nursing professionals.

## 1. Introduction

Seasonal influenza, caused by influenza viruses, is a global issue that affects approximately 1 billion people each year [1]. Unlike avian influenza and the influenza pandemic, seasonal influenza is a common acute respiratory tract infection [2]. It can be rapidly and easily transmitted through droplets or by direct contact with the secretions of infected individuals [1,2]. While most individuals recover in a few days or up to two weeks without treatment or complications [2], there are 3–5 million severe respiratory illnesses and 290,000 to 650,000 respiratory deaths annually [1]. Through the development of the Global Influenza Programme and the Global Influenza Surveillance and Response System in the 1950s, the WHO began to support and enhance influenza response competencies at the national, regional, and global levels. It now provides prompt evidence for national policies on antiviral use, further strengthening influenza vaccine protection for at-risk or vulnerable populations [1].

Seasonal influenza vaccination is a safe and effective measure for preventing infection and its related complications, especially for high-risk groups, including healthcare workers [1,2,3]. To safeguard the efficacy, quality, and safety of influenza vaccines, the WHO provides guidelines and recommendations to support producers in their development and manufacturing [3]. However, vaccine hesitancy has been a significant challenge for many countries over the past decade [4]. Various factors contribute to this hesitancy, including concerns about vaccine safety, misinformation, myths [4], and vaccine literacy [5]. Common perceived barriers include the inconvenience of receiving the vaccine, doubts about its effectiveness, and apprehensions about potential side effects [6]. In addition, many countries in the WHO South-East Asian Region lack effective, widespread seasonal influenza vaccination strategies, resulting in low local vaccination rates [7].

HCPs are at significant risk of contracting influenza through patient interactions required by the role, which can also lead to the virus spreading to at-risk individuals [8]. Though vaccination helps safeguard both HCPs and those in their vicinity [1,3], evidence on healthcare workers’ perceptions of influenza vaccination differs. Despite international and national support, several systematic reviews of studies and pieces of evidence have shown that seasonal influenza vaccine acceptance among HCPs worldwide has remained variable, influenced by different factors over the past few decades [9,10,11,12].

For instance, Gualano et al. [9] reviewed 52 studies conducted from 2000 to 2019 across different continents on HCPs’ attitudes towards mandatory influenza vaccination. The pooled results showed that 61% of HCPs agreed with the influenza vaccination policy, with substantial variation across continents (Europe, 54%; Asia, 69%) and professional groups (nurses, 48%; students, 80%). Vaccinated HCPs were more likely to support mandatory vaccination than unvaccinated HCPs. Additionally, Fan et al. [12] examined 92 studies from 26 countries regarding HCPs’ influenza vaccination rates. The overall influenza vaccination rate was low (41.7%), with significant disparities across regions (Americas, 67.1%; Europe, 42.5%; and Africa, 6.5%) and between low-income and high-income countries (46.9% and 35.6%, respectively). The determinants associated with vaccination were age, length of employment, education level, working department, career, awareness of the risk of infection, and vaccines. Similarly, a global review of 368 studies on healthcare providers’ attitudes toward influenza vaccination revealed that they are influenced by intrinsic and extrinsic motivations, organizational dynamics, and policy considerations [13].

In recent years, evidence has shown that concerns about COVID-19 may have contributed to changes in influenza vaccination among healthcare professionals (HCPs) [14,15]. The COVID-19 pandemic enhanced global intentions to be vaccinated against influenza. For example, Bianchi et al. [14] reviewed the impact of the COVID-19 pandemic on flu vaccination rates in 14 European studies published from 2021 to 2023. The review reported a significant association between flu vaccine uptake and COVID-19-related factors, including fear of coronavirus infection, COVID-19 vaccination, and COVID-19 and influenza symptoms. Greater influenza vaccination coverage, rising from 17% to 38%, was reported in 2020/2021 compared to the previous year, driven by increased awareness of influenza vaccination.

Nursing students are future nursing professionals and have a duty to disseminate and promote health education, including with respect to vaccination and vaccine-preventable diseases, among patients and the community. Studies have shown that influenza vaccine uptake among nursing students has ranged from 19.1 to 37.65% in the recent decade across various countries; for instance, it was 19.1% [16] and 23% [17] in Greece, 30.7% in Poland [18], and 37.65% in Italy [19]. Nursing students showed greater hesitation towards vaccination than practice nurses, particularly regarding the long-term effects of vaccines [20]. However, individual confidence in vaccines can differ. This hesitancy may indicate differences in vaccine confidence and acceptance.

Hong Kong is a tiny yet densely populated city that had more than 7.5 million residents in mid-2025 [21]. Preventive measures taken to minimize transmission of influenza to high-risk or vulnerable groups are crucial. The HKSAR government encourages all citizens to engage in seasonal influenza vaccination for personal protection. The government provides free or subsidized annual seasonal influenza vaccination through various service providers to eligible persons—e.g., pregnant women and persons aged 50 or above with chronic illnesses and regular follow-up appointments [22]—and high-risk populations, e.g., healthcare workers, residents of Residential Care Homes, and poultry workers [23]. Eligible persons and high-risk populations can be vaccinated with influenza vaccines at public clinics, District Health Centers, etc., or by private doctors participating in the Vaccination Subsidy Scheme, with a subsidy of about HKD 260 (~USD 33) per dose [24]. In 2024, the price of vaccination was ~HKD 250 (~USD 30) [25] or higher, depending on the type of service and the service provider [26]. Although influenza vaccination is not currently mandatory for HCPs in Hong Kong, the Scientific Committee on Vaccine Preventable Diseases (SCVPD) strongly recommends that they get vaccinated to minimize morbidity and work absenteeism [27]. In contrast, university students enrolled in healthcare programs are not eligible for free or subsidized seasonal influenza vaccination because they are not yet healthcare workers. Nevertheless, most local tertiary institutions cannot offer free seasonal influenza vaccination to nursing students every year.

Evidence has shown that influenza vaccination uptake among local nurses in clinical and long-term care facilities is low, ranging from 32% to 45% from 2014 to 2018 [28,29]. Lau et al. [28] reported that most local nurses (66%) had moderate or high levels of influenza vaccine hesitancy before the COVID-19 pandemic. In 2020, Wang et al. [30] examined the impact of the pandemic on influenza vaccination intention and acceptance among 806 nurses. They found that more nurses shifted from refusal to hesitancy or acceptance, and those with suspected or confirmed cases of COVID-19 were more likely to accept the influenza vaccine [30].

About a decade ago, Cheung et al. [31] assessed influenza vaccination uptake rates and willingness to receive an annual seasonal influenza vaccine among 902 local nursing students using the Health Belief Model (HBM). A very low uptake rate (15.2%) and associations with perceived susceptibility, perceived seriousness, and perceived barriers were found. Nursing students also have a professional responsibility to protect patients’ safety and their own health through influenza vaccination. To determine whether the uptake rate of influenza vaccination has increased over the years after the COVID-19 pandemic, we examined uptake rates of the seasonal influenza vaccine and the predictors of Hong Kong nursing students’ willingness to receive it.

## 2. Materials and Methods

### 2.1. Study Design

We adopted a quantitative, descriptive, cross-sectional study design, which is an efficient and inexpensive method whereby researchers can collect data from a target population at a specific time [32]. It is commonly used to examine factors affecting health, understand health-related outcomes, and characterize population characteristics.

### 2.2. Participants

The target participants in this study were Hong Kong university students aged 18 or older who were enrolled in undergraduate general nursing programs and able to understand English, as it was the language used in the questionnaire. Students enrolled in mental health nursing programs were excluded because of differences in professional roles and exposure risks.

### 2.3. Sampling

The nursing student participants were recruited using convenience sampling from a large, self-financing non-profit university that offers both undergraduate and postgraduate programs. It enrolls the largest number of general undergraduate nursing students (~2500 in 2024) in Hong Kong.

For sample size calculation, the sample size requirement for the logistic regression analysis was determined using the Events-Per-Variable (EPV) criterion to ensure stable parameter estimation. In accordance with the methodological standard of EPV ≥ 10 [33] and planning for 10 predictors and an estimated intention to vaccinate of 30%, we set the minimum total sample size to 334.

### 2.4. Instrument and Outcome Measures

The self-completed online questionnaire used in this study was divided into three parts. The first part included 11 items collecting demographic information from the participants: program, year of study, gender, age, marital status, seasonal influenza vaccination history, frequency of vaccination over the past five years, intentions regarding vaccination in the coming year, perceptions of reasonable cost, history of COVID-19 infection, and the number of times vaccinated with COVID-19 vaccines.

The second and third parts, “Nursing Students Seasonal Influenza (Flu) Survey,” were adopted from a previous local study with the author’s permission [31]. The survey was written in English and developed based on the Health Belief Model to allow investigation of influenza vaccination. The Health Belief Model (HBM) is a theoretical framework developed in the 1950s by social psychologists to understand the factors influencing individuals’ decisions to engage in health-promoting behaviors [34]. The Health Belief Model is one of the most commonly used psychological models for predicting influenza vaccination uptake among HCPs [34,35]. This survey was rigorously validated by four experts, achieving a content validity index of 0.96 [31]. Its test–retest reliability was assessed by 10 nursing students, yielding a reliability of 0.94. Also, the internal consistency and reliability of the subscales ranged from 0.55 to 0.84 [31].

The second part consisted of fifty-six items organized into seven subdomains based on the Health Belief Model. The subdomains were perceived susceptibility, perceived seriousness, perceived benefits, perceived barriers, knowledge, cue to action, and health motivation. This section was designed to assess the participants’ perceptions of seasonal influenza. Responses were measured using a four-point Likert scale, ranging from “strongly disagree” (1) to “strongly agree” (4). Higher overall scores across subdomains indicated stronger agreement with the corresponding statements.

The third part included four questions regarding participants’ health information, focusing on self-perceived health status, any chronic diseases, frequency of medical consultations in the past year, and occurrences of flu-like symptoms within the previous six months.

As the “Nursing Students Seasonal Influenza (Flu) Survey” was developed years ago, before the COVID-19 pandemic, a minor addition, i.e., the inclusion of “COVID-19”, to the wording of the following item was made with the author’s permission: “The incident of SARS, Swine Flu, or COVID-19 has promoted me to get a flu shot.” This modification was necessary in order to allow the questionnaire to better reflect current health concerns, particularly regarding post-COVID-19 vaccination intentions. To assess the feasibility and appropriateness of the online questionnaire, 10 nursing students were invited to participate in a pilot test, with completion time and feedback collected. The pilot participants reported that the English questionnaire was relevant, appropriate, and easy to understand, with no further amendments made. To assess this study’s reliability, we adapted the Health Belief Model subdomains from Cheung et al.’s study [31], and Cronbach’s alpha for the 7 subdomains ranged from 0.68 to 0.92.

### 2.5. Ethical Considerations

Before this study was conducted, ethical approval was obtained from the Research and Ethics Committee of Saint Francis University (Reference No. SEWG/HP/2024/group 40). This research was conducted in accordance with the Declaration of Helsinki. All potential participants received information about the study, including its aims, risks, benefits, procedures, and the voluntary nature of participation, via an online information sheet. If they agreed to participate, they provided their electronic consent and completed the online questionnaire. Participants could decline to answer specific questions or withdraw from the survey at any time without consequences. To guarantee participants’ anonymity, all data were kept confidential. Only the research team members were authorized to access the data. The electronic database file will be deleted 5 years after the data collection period.

### 2.6. Data Collection and Analysis

After ethical approval was received, data collection took place from early November to early December 2024. Two recruitment methods were used (with permission): face-to-face invitations to nursing students attending classes on the university campus, and email invitations were sent to nursing students in clinical placements at hospitals; both invitations asked the participants to complete online surveys. The researchers explained the details of the study to the nursing students after class and invited them to participate, answering any questions they had. Nursing students on practicum received mass email invitations to participate and were encouraged to reach out to the researchers if they had any questions. Of the approximately 2500 targeted participants at the university, 542 started the online survey, and 461 completed it (81 surveys were incomplete and thus excluded). The response rate was about 18.4%.

The sociodemographic characteristics of the participants were summarized using descriptive statistics. Categorical variables were expressed as frequencies and percentages [N (%)], and normally distributed continuous variables were reported as means ± standard deviations (SDs). Group comparisons for continuous parametric variables were performed using independent-samples t-tests. For categorical variables, the Pearson chi-square test or Fisher’s exact test was applied, as appropriate, to evaluate statistical significance. Variables with a *p*-value < 0.1 in univariate analyses were subsequently entered into a logistic regression model to identify any significant factors. All statistical analyses were conducted using IBM SPSS Statistics for Windows, Version 26.0 (IBM Corp., Armonk, NY, USA), with significance set at a two-tailed *p*-value of <0.05.

## 3. Results

Table 1 details the demographic and health profiles of the study cohort, comprising 461 participants. The sample was predominantly female (67.5%), with a mean age of 20.53 (SD = 2.16) years, and nearly all were single (98.9%). The majority were students enrolled in the Bachelor of Nursing (Honors) program (96.1%), with a notable concentration in the fourth academic year (38.2%). Vaccination history was diverse: 34.3% reported undergoing influenza vaccination during the most recent year. In the preceding five years, 49.7%, 11.5%, and 5% underwent vaccination 1–2 times, 3–4 times, and annually, respectively. Furthermore, 49.2% and 25.8% of the participants agreed that the reasonable cost of one instance of influenza vaccination should be zero (free of charge) or below HKD 100 (~USD 13), respectively. Regarding their COVID-19 statuses, 85.2% reported having a prior infection, and 69.2% had completed a three-dose vaccination regimen. Most of the participants self-assessed their health status as fair to good (84.4%). Also, 45.3% of the participants reported that they had not visited a physician in the past 12 months, and 56.3% experienced at least one episode of flu-like symptoms in the past 6 months. Assessment of knowledge and Health Belief Model (HBM) domains revealed a mean score of 2.87 (SD = 0.47) for knowledge and 2.51 (SD = 0.55) for perceived susceptibility.

Table 2 displays the univariate analysis examining the association between various demographic and clinical factors and the participants’ intention to undergo influenza vaccination in the coming year. No significant associations were observed for any of the demographic variables (program enrollment, year of study, gender, age, or marital status). However, intention to get vaccinated was significantly associated with prior vaccination behavior (*p* < 0.001). Those planning to get the shot had a substantially higher rate of vaccination in the last season (59.3% vs. 22.8%) (*p* < 0.001) and reported a greater frequency of past vaccinations over the preceding five years (*p* < 0.001). A significant association with physician consultation frequency was also found, where participants intending to get vaccinated reported more consultations in the previous 12 months (*p* = 0.007). Furthermore, all Health Belief Model (HBM) constructs were significantly higher for those intending to get vaccinated, including perceived susceptibility (*p* < 0.001), perceived seriousness (*p* = 0.001), perceived benefits (*p* < 0.001), cue to action (*p* < 0.001), and health motivation (*p* = 0.001).

Table 3 presents the results of the logistic regression analysis identifying the factors affecting intention to undergo influenza vaccination in the coming year. Participants who had undergone influenza vaccination in the last flu season were more likely to report that they intended to get vaccinated (OR = 2.881, 95% CI: 1.773–4.680, *p* < 0.001). This strong association persisted when examining the frequency of vaccination over the preceding five years: compared to those who had never been vaccinated, those who had been vaccinated one to two times had an OR of 3.239 (95% CI: 1.750–5.993, *p* < 0.001), those who had been vaccinated three to four times had an OR of 3.984 (95% CI: 1.773–8.957, *p* < 0.001), and those who got vaccinated every year had an OR of 10.353 (95% CI: 3.025–35.436, *p* < 0.001).

Of the Health Belief Model (HBM) constructs, perceived susceptibility was the only significant independent predictor (OR = 2.244, 95% CI: 1.302–3.867, *p* = 0.004). Physician consultation frequency (past 12 months) and the remaining HBM constructs (perceived seriousness, perceived benefits, cue to action, and health motivation) were not found to be significant factors affecting vaccination intention in the regression model.

## 4. Discussion

In our study, approximately one-third (34.3%) of the nursing student participants reported that they had undergone seasonal influenza vaccination in the most recent season in 2024. This figure is higher than that reported in Cheung et al.’s study from about a decade ago [31], which investigated 902 nursing students using the same measurement scale, reporting 15.2%. Compared with uptake rates among nursing students in recent studies from other countries, our result is slightly lower; consider, for instance, the value reported in an Italian study: 37.65% [19]. However, our value is higher than values reported in Poland (30.7%) [18] and Greece, where the rates were 23% [17] and 19.1% [16], respectively. Though our study’s results show an improvement in local uptake rates, they remain unsatisfactory compared with the overall vaccination rate (41.7%) among HCPs reported in a systematic review of 92 studies [12].

Nursing students are future nursing professionals who must fulfill a clinical practicum requirement before graduation [36]. They are also obligated to protect patients’ safety and their own health through influenza vaccination. As mentioned, university nursing students are not eligible for free or subsidized seasonal influenza vaccination, and not all local tertiary institutions offer vaccination to nursing students every year. The price of vaccination was once ~HKD 250 (~USD 30) [25] or above, depending on the service provider [26]. This financial burden might explain why most of our student participants expected influenza vaccination to cost less than HKD 100 (~USD 13) (25.8%) or even be free (49.2%), and the low uptake rates (34.3%) might stem from this issue as well. It has been suggested that, along with healthcare workers, the Hong Kong government should also consider all future HCPs, i.e., healthcare students, to be a priority group for influenza vaccination [22,23]. In addition, by collaborating with the local government or healthcare organizations, universities with healthcare programs may explore the feasibility of implementing a free, easily accessible, and comprehensive influenza vaccination program for on-campus healthcare students. In contrast, although the literature suggests that mandatory influenza vaccination results in higher uptake rates among nursing students (89.3%) relative to other university students (45.8–79.8%) [37], there are conflicting views regarding mandatory vaccination [9,38]. Careful consideration, along with adequate consultation and discussion with HCPs and students about compulsory influenza vaccination, is recommended for local governments and universities.

In total, 61.2% of our participants had been vaccinated against influenza one to four times over the past five years, while only 5% were vaccinated annually (Table 1). These percentages are higher than those reported by Cheung et al. [31], namely, 41.8% and 2.1% for the same categories. Though 31.5% of our participants planned to undergo influenza vaccination in the coming year, 44.3% were still hesitant or unsure (Table 1). Additionally, intention to get vaccinated in the coming year was significantly associated with the participants’ prior vaccination behavior (*p* < 0.001), including vaccination in the most recent year and a greater frequency of vaccinations over the past five years (Table 2). Pinatel et al. [11] reviewed 11 qualitative studies published between 2009 and 2020, focusing on factors influencing nurses’ vaccination against influenza and their hesitancy in this regard. The key determinants included the concepts of risk and the duty to maintain health, misunderstood immunological beliefs, and institutional and hierarchical hesitancy, which encompasses inadequate information and difficulty accessing healthcare. Martínez-Serrano et al. [39] recently systematically reviewed 38 studies on methods for enhancing vaccination acceptance, including community education, tailored messages, media, and new technology. The most effective strategies for reducing hesitancy include multi-aspect, well-organized educational interventions designed for specific groups [39]. For instance, early education programs for nursing students on influenza and the benefits of vaccination [17] and a healthcare-student-led vaccination promotion campaign that highlighted such workers’ professional responsibility to vaccinate were found to have a positive influence on vaccination acceptance [40]. Local governments, healthcare organizations, and university faculties should work together to provide timely, clear information; clarify misunderstandings of concepts; and facilitate access to healthcare services, thereby improving uptake rates.

Approximately half of our participants (50.4%) rated their health as either good (43%) or excellent (7.4%). These results are consistent with the findings of Cheung et al. [31], who reported that 46.4% of their participants rated their health as good, while 3.7% reported that it was excellent. Notably, the fact that half of our participants reported good health aligns with their relatively low need for medical visits in the past year: 45.3% reported not requiring a physician’s visit, and 39.9% reported visiting a physician only 1–2 times. Additionally, we found a significant association between the participants’ intention to get vaccinated and the number of medical consultations they had taken part in the previous 12 months (*p* = 0.007). Although most Health Belief Model (HBM) constructs— including perceived susceptibility, perceived seriousness, perceived benefits, cue to action, and health motivation—were significantly higher for those intending to get vaccinated in our study, perceived susceptibility was the only significant independent predictor of vaccine intention in our regression model. This finding could indicate that the nursing student participants believed they were at high risk of acquiring seasonal influenza and were more likely to get vaccinated than others who did not feel vulnerable. The more susceptible they felt, the more likely they were to engage in preventive health behaviors. This finding aligns with the results of a previous local study, in which nursing professionals viewed influenza as a threat to their own health [41].

In our study, most of the participants (85.2%) had previously contracted COVID-19 and received three or more doses of the COVID-19 vaccine (72.7%) before the data were collected in 2024. Although we did not observe any significant associations between intention to undergo influenza vaccination in the coming year and COVID-19 infection or vaccination doses, the pandemic may have been a turning point and a valuable lesson for increasing influenza vaccination and reducing vaccine hesitancy. Indeed, the COVID-19 pandemic had overwhelming impacts on the health of communities and populations globally and locally [42,43,44,45,46], including HCPs and nursing students [47,48]. Local undergraduate nursing students were greatly afraid of infection, had negative emotional states, and exhibited substantially impacted quality of life during the pandemic [48]. Evidence indicated that fear of contracting COVID-19 increased global willingness and intention to undergo influenza vaccination across continents, including Asia, Europe, and North America [15]. The chief motivator was the perceived benefit of safeguarding oneself and others against influenza, while barriers to uptake included concerns about vaccine effectiveness and side effects [15].

The Hong Kong government, healthcare organizations, and universities are encouraged to collaboratively develop effective strategies and provide adequate support to promote annual seasonal influenza vaccination for all current and future HCPs, including local nursing students. Vaccinating staff not only protects them but also reduces the risk of influenza being transmitted to high-risk, vulnerable patients who are at risk of severe illness and complications. We also recommend that parties behind future policy reform and practice consider ensuring that appropriate health information systems are designed and in place to track healthcare workers’ vaccination records. To reduce the incidence of seasonal influenza, Haider and Hassan [7] suggested implementing context-specific influenza vaccination policies, improving public education, and enhancing surveillance at both the national and international levels, thereby strengthening pandemic preparedness and responses in the near future. Additionally, strategies should be comprehensive and flexible to enhance influenza vaccination rates and improve public health outcomes [13].

A recent study reported that a strong sense of professional responsibility, confidence in the vaccines, and a desire to protect both oneself and one’s patients were key factors influencing HCPs’ influenza vaccination intentions and behavior and that offering free, easily accessible vaccination services can translate intention to get vaccinated into behavior [49]. Specialized training could enable future professionals to become knowledgeable and trustworthy advocates for vaccination [50]. Nursing faculty are ideally positioned to provide current information about influenza, the benefits of vaccination, and free vaccination coverage prior to clinical placement. Faculty members should consider integrating this information to better educate students, encourage proactive measures for improving infection prevention and control practices, and increase demand for the vaccine. Ultimately, this approach will help cultivate a culture of vaccination among future healthcare professionals and foster a sense of professional responsibility to protect public health. By ensuring that citizens are well-informed and vaccinated, we can help prevent influenza outbreaks and promote overall public health.

This study has several limitations. We used convenience sampling, which did not ensure adequate representation. This single-institution approach raises concerns about potential bias and limits the generalizability of the findings. In 2024, there were approximately 10,000 university undergraduate nursing students in Hong Kong. This non-profit, self-financed university offers a diverse range of nursing programs and enrolls the largest number of general undergraduate nursing students in the region, with approximately 2500 enrolled in 2024. Although its student demographics may not fully represent the overall population, the insights gained provide direction for improving vaccination rates. A future multi-site study could adopt a more appropriate random sampling method to include nursing students from various educational settings, thereby minimizing potential sampling bias.

Online surveys offer several advantages, such as saving time and money; however, they also present challenges, including concerns about data validity and the reliability of self-assessed responses [51]. Online self-report questionnaires can lead to reporting bias or misinterpretation of questions. In this study, potential participants were provided with a QR code linked to the questionnaire in their classrooms after class or received an email invitation to join the study voluntarily. They had opportunities to ask questions, seek clarification on any misunderstandings, or contact the researchers.

Moreover, self-reporting and recall bias regarding vaccination and health history may generate significant concerns and introduce significant limitations for this study. Self-reporting surveys may introduce social desirability or response bias, which can undermine the validity and accuracy of the measurements [52]. Relying on participants’ memory could lead to inaccuracies rather than valid clinical data with which to confirm vaccination histories.

Ultimately, this study’s cross-sectional design limits causal interpretation and the understanding of long-term trends in influenza vaccination uptake and the evolving characteristics of the study population. We recommend conducting a multi-site longitudinal study to track the conversion from intention to behavior and evaluate interventions informed by the Health Belief Model (HBM). Additionally, using mixed methods to explore participants’ hesitancy and confidence regarding vaccination uptake would be beneficial.

## 5. Conclusions

Annual seasonal influenza vaccination rates among nursing students remain low, though they have improved in recent years. Factors related to intention to undergo influenza vaccination among nursing students in the coming year included prior vaccination behavior and perceived susceptibility to seasonal influenza. Given the results on nursing students’ influenza vaccination behaviors and vaccination intentions, the Hong Kong government should also consider including nursing students as a priority group for influenza vaccination, along with healthcare workers, and offer free or less expensive vaccination. Healthcare organizations should develop reliable health information systems to monitor the vaccination statuses of healthcare professionals to ensure the safety of both patients and staff. Additionally, universities with healthcare programs might consider offering free influenza vaccination clinics for their students and incorporating influenza-related information into curricula to improve students’ education, promote proactive approaches to infection prevention and control, and boost vaccination rates. Further in-depth research into the reasons behind vaccine hesitancy and barriers to vaccination, or a longitudinal study to evaluate the transition from vaccination intention to behavior, is also encouraged.

## Figures and Tables

**Table 1:** Demographics and Health Profiles of the participants (*N* = 461).

Demographics	Mean ± SD/N (%)
Program enrolled in	
Bachelor of Nursing (Honors) (BNUR)	443 (96.1%)
Bachelor of Nursing (Honors) (Applied Degree Places) (BNAD)	18 (3.9%)
Year of study	
First year	130 (28.2%)
Second year	61 (13.2%)
Third year	15 (3.3%)
Fourth year	176 (38.2%)
Fifth year	79 (17.1%)
Gender	
Male	150 (32.5%)
Female	311 (67.5%)
Age	20.53 ± 2.16
Marital status	
Single	456 (98.9%)
Married	5 (1.1%)
Have you received an influenza vaccination during the most recent year?	
Yes	158 (34.3%)
No	303 (65.7%)
How many times have you received an influenza vaccination in the past 5 years?	
I have not received a vaccination	156 (33.8%)
1–2 times	229 (49.7%)
3–4 times	53 (11.5%)
I have received a vaccination every year	23 (5.0%)
Will you plan on getting an influenza vaccination when it is available in the coming year?	
No	112 (24.3%)
Yes	145 (31.5%)
Not sure	204 (44.3%)
The reasonable cost for one influenza vaccination should be:	
Free of charge	227 (49.2%)
Below HKD 100	119 (25.8%)
HKD 100–300	107 (23.2%)
More than HKD 300	8 (1.7%)
Have you ever had COVID-19 before?	
Yes	393 (85.2%)
No	68 (14.8%)
How many doses of the COVID-19 vaccine have you received?	
0 doses	4 (0.9%)
1 dose	16 (3.5%)
2 doses	106 (23.0%)
3 doses	319 (69.2%)
4 doses or above	16 (3.5%)
How would you rate your health?	
Poor	38 (8.2%)
Fair	191 (41.4%)
Good	198 (43.0%)
Excellent	34 (7.4%)
How many times did you visit your physician in the past 12 months?	
Zero	209 (45.3%)
1–2	184 (39.9%)
3–5	46 (10.0%)
More than 5	22 (4.8%)
How many times have you had flu/flu-like symptoms (chills, cough, fever, sore throat) in the past 6 months? (N = 460)	
Zero	201 (43.7%)
1 to 3	213 (46.3%)
More than 3	46 (10.0%)
Health Belief Model (6 domains) + Knowledge (mean, SD)	
Knowledge	2.87 ± 0.47
Perceived susceptibility	2.51 ± 0.55
Perceived seriousness	2.80 ± 0.57
Perceived benefits	2.74 ± 0.58
Perceived barriers	2.46 ± 0.57
Cue to action	2.47 ± 0.66
Health motivation	2.65 ± 0.54

**Table 2:** Univariate analysis of factors associated with intending to get vaccinated against influenza in the coming year.

	Not Planning to Undergo Influenza Vaccination in the Coming Year/Not Sure (N = 316)	Planning to Undergo Influenza Vaccination in the Coming Year (N = 145)		
Demographics	Mean ± SD/N (%)	Mean ± SD/N (%)	OR (95% CI of OR)	*p*-value
Program enrolled in				0.861 ^c^
Bachelor of Nursing (Honors) (BNUR)	304 (95.9%)	139 (95.9%)	1	
Bachelor of Nursing (Honors) (Applied Degree Places)	12 (3.8%)	6 (4.1%)	1.094 (0.402–2.973)	
Year of study				0.187 ^c^
First year	87 (27.5%)	43 (29.7%)	1	
Second year	43 (13.6%)	18 (12.4%)	0.847 (0.438–1.639)	
Third year	11 (3.5%)	4 (2.8%)	0.736 (0.221–2.446)	
Fourth year	129 (40.8%)	47 (32.4%)	0.737 (0.449–1.209)	
Fifth year	46 (14.6%)	33 (22.8%)	1.451 (0.815–2.586)	
Gender				0.213 ^c^
Male	97 (30.7%)	53 (36.6%)	1.301 (0.860–1.968)	
Female	219 (69.3%)	92 (63.4%)	1	
Age	20.54 ± 2.16	20.50 ± 2.17	0.990 (0.903–1.085)	0.826 ^t^
Marital status				0.182 ^f^
Single	314 (99.4%)	142 (97.9%)	1	
Married	2 (0.6%)	3 (2.1%)	3.317 (0.548–20.069)	
Have you received an influenza vaccination during the most recent year?				* <0.001 ^c^
Yes	72 (22.8%)	86 (59.3%)	4.940 (3.236–7.540)	
No	244 (77.2%)	59 (40.7%)	1	
How many times have you received an influenza vaccination in the past 5 years?				* <0.001^c^
I have not received a vaccination	139 (44.0%)	17 (11.7%)	1	
1–2 times	142 (44.9%)	87 (60.0%)	5.010 (2.833–8.857)	
3–4 times	30 (9.5%)	23 (15.9%)	6.269 (2.989–13.148)	
I have received a vaccination every year	5 (1.6%)	18 (12.4%)	29.435 (9.687–89.446)	
The reasonable cost for one vaccination should be:				0.099 ^c^
Free of charge	154 (48.7%)	73 (50.3%)	1	
Below HKD 100	89 (28.2%)	30 (20.7%)	0.711 (0.432–1.171)	
HKD 100–300	70 (22.2%)	37 (25.5%)	1.115 (0.686–1.813)	
More than HKD 300	3 (0.9%)	5 (3.4%)	3.516 (0.818–15.112)	
Have you ever had COVID-19 before?				0.338 ^c^
Yes	266 (84.2%)	127 (87.6%)	1.326 (0.743–2.366)	
No	50 (15.8%)	18 (12.4%)	1	
How many doses of the COVID-19 vaccine have you received?				0.428 ^f^
0 doses	3 (0.9%)	1 (0.7%)	0.429 (0.036–5.063)	
1 dose	9 (2.8%)	7 (4.8%)	1.000 (0.247–4.042)	
2 doses	78 (24.7%)	28 (19.3%)	0.462 (0.157–1.356)	
3 doses	217 (68.7%)	102 (70.3%)	0.604 (0.219–1.668)	
4 doses or above	9 (2.8%)	7 (4.8%)	1	
How would you rate your health?				0.293 ^c^
Poor	30 (9.5%)	8 (5.5%)	0.381 (0.135–1.074)	
Fair	128 (40.5%)	63 (43.4%)	0.703 (0.333–1.484)	
Good	138 (43.7%)	60 (41.4%)	0.621 (0.294–1.311)	
Excellent	20 (6.3%)	14 (9.7%)	1	
How many times did you visit your physician in the past 12 months?				*0.007 ^c^
Zero	160 (50.6%)	49 (33.8%)	1	
1–2	114 (36.1%)	70 (48.3%)	2.005 (1.295–3.104)	
3–5	30 (9.5%)	16 (11.0%)	1.741 (0.877–3.458)	
More than 5	12 (3.8%)	10 (6.9%)	2.721 (1.108–6.680)	
How many times have you had flu/flu-like symptoms (chills, cough, fever, sore throat) in the past 6 months? (*N* = 460)				0.458 ^c^
Less than 1	138 (43.8%)	63 (43.4%)	1	
1 to 3	142 (45.1%)	71 (49.0%)	1.095 (0.725–1.654)	
More than 3	35 (11.1%)	11 (7.6%)	0.688 (0.328–1.443)	
Health Belief Model (6 domains) + Knowledge (mean, SD)				
Knowledge	2.85 ± 0.47	2.91 ± 0.47	1.328 (0.867–2.034)	0.192 ^t^
Perceived susceptibility	2.41 ± 0.53	2.73 ± 0.55	3.218 (2.138–4.843)	* <0.001 ^t^
Perceived seriousness	2.74 ± 0.55	2.93 ± 0.56	1.814 (1.253–2.627)	* 0.001 ^t^
Perceived benefits	2.66 ± 0.59	2.91 ± 0.54	2.189 (1.501–3.193)	* <0.001 ^t^
Perceived barriers	2.47 ± 0.55	2.43 ± 0.60	0.862 (0.607–1.223)	0.405 ^t^
Cue to action	2.37 ± 0.67	2.71 ± 0.59	2.325 (1.675–3.229)	* <0.001 ^t^
Health motivation	2.60 ± 0.53	2.78 ± 0.54	1.930 (1.314–2.834)	* 0.001 ^t^

* *p* < 0.05. ^t^—independent-samples *t* test. ^c^—Pearson Chi-square test. ^f^—Fisher’s Exact test. OR—Odds Ratio. CI—Confidence Interval.

**Table 3:** Logistic regression identifying the factors related to intention to undergo influenza vaccination in the coming year (Yes or No only).

Variables	OR (95% CI of OR)	*p*-Value
Underwent influenza vaccination last year	1.645 (0.839–3.223)	0.147
Number of times vaccinated against influenza in the past five years		
I have not undergone vaccination	1	
1–2 times	5.156 (2.436–10.917)	* <0.001
3–4 times	8.220 (2.683–25.180)	* <0.001
I get vaccinated every year	11.530 (2.063–64.436)	* 0.005
Number of times you visited your physician in the past 12 months		
Zero	1	
1–2	1.657 (0.843–3.257)	0.143
3–5	1.356 (0.469–3.915)	0.574
More than 5	2.911 (0.540–15.687)	0.214
Perceived susceptibility	3.076 (1.569–6.033)	* 0.001
Perceived seriousness	1.123 (0.569–2.217)	0.737
Perceived benefits	1.250 (0.618–2.526)	0.535
Cue to action	1.970 (1.026–3.782)	0.042
Health motivation	0.525 (0.235–1.170)	0.115

* Significant result according to the Bonferroni correction adjustment method. OR—Odds Ratio. CI—Confidence Interval. Remark: If all variables in Table 2 (i.e., Full model) are included, the Akaike Information Criterion (AIC) = 479.09, and the area under the curve (AUC) = 0.838 (95% CI = 0.798–0.879). If the model includes variables for which *p* < 0.1 (Table 3), AIC = 453.109 and AUC = 0.794 (95% CI = 0.751–0.838).

## Data Availability

The data presented in this study are available upon reasonable request from the corresponding author. They are not publicly available due to privacy or ethical restrictions.

## References

[1] World Health Organization (2025). Influenza (Seasonal). https://www.who.int/news-room/fact-sheets/detail/influenza-(seasonal).

[2] Centre for Health Protection of the Hong Kong Special Administrative Region (2024). Seasonal Influenza. https://www.chp.gov.hk/en/healthtopics/content/24/29.html.

[3] World Health Organization Vaccine Against Influenza: WHO Position Paper—May 2022. https://www.who.int/publications/i/item/who-wer9719.

[4] World Health Organization Vaccine Hesitancy: A Growing Challenge for Immunization Programmes. https://www.who.int/news/item/18-08-2015-vaccine-hesitancy-a-growing-challenge-for-immunization-programmes.

[5] Zhang E., Dai Z., Wang S., Wang X., Zhang X., Fang Q. (2023). Vaccine literacy and vaccination: A systematic review. Int. J. Public Health.

[6] Lamot M., Kirbiš A. (2025). Seasonal influenza vaccination uptake among health and medical college students: A discrete choice experiment. Hum. Vaccin. Immunother..

[7] Haider S., Hassan M.Z. (2025). Seasonal influenza surveillance and vaccination policies in the WHO South-East Asian Region. BMJ Glob. Health.

[8] Centers for Disease Control and Prevention Influenza (Flu). Clinical Guidance for Influenza. https://www.cdc.gov/flu/hcp/clinical-guidance/index.html.

[9] Gualano M.R., Corradi A., Voglino G., Catozzi D., Olivero E., Corezzi M., Bert F., Siliquini R. (2021). Healthcare Workers’(HCWs) attitudes towards mandatory influenza vaccination: A systematic review and meta-analysis. Vaccine.

[10] Ledda C., Motta G., Rapisarda V., Maltezou H.C. (2023). Influenza immunization of healthcare personnel in the post-COVID-19 pandemic era: Still a lot to do!. Vaccine.

[11] Pinatel N., Plotton C., Pozzetto B., Gocko X. (2022). Nurses’ influenza vaccination and hesitancy: A systematic review of qualitative literature. Vaccines.

[12] Fan J., Xu S., Liu Y., Ma X., Cao J., Fan C., Bao S. (2023). Influenza vaccination rates among healthcare workers: A systematic review and meta-analysis investigating influencing factors. Front. Public Health.

[13] Dardas L.A., Al-Leimon O., Jaber A.R., Saadeh M., Al-Leimon A., Al-Hurani A., Jaber A.-R., Aziziye O., Al-salieby F., Aljahalin M. (2023). Flu shots unveiled: A global systematic review of healthcare providers’ uptake of, perceptions, and attitudes toward influenza vaccination. Vaccines.

[14] Bianchi F.P., Cuscianna E., Rizzi D., Signorile N., Daleno A., Migliore G., Tafuri S. (2023). Impact of COVID-19 pandemic on flu vaccine uptake in healthcare workers in Europe: A systematic review and meta-analysis. Expert Rev. Vaccines.

[15] Kong G., Lim N.A., Chin Y.H., Ng Y.P.M., Amin Z. (2022). Effect of COVID-19 pandemic on influenza vaccination intention: A meta-analysis and systematic review. Vaccines.

[16] Tseroni M., Tsavdaroglou T., Galanis P., Mangoulia P., Velonaki V.S., Sourtzi P. (2024). Vaccination Coverage Among Nursing Students in Greece: A Cross-Sectional Assessment. Preprint.

[17] Statiri A., Adamakidou T., Margari N., Govina O., Tsiou C., Giakoumidakis K., Dokoutsidou E. (2024). Influenza vaccination of nursing students: A cross-sectional study of uptake, knowledge, attitudes, and practices in Greece. Diseases.

[18] Kałucka S., Dziankowska-Zaborszczyk E., Grzegorczyk-Karolak I., Głowacka A. (2020). A comparison of the attitudes to influenza vaccination held by nursing, midwifery, pharmacy, and public health students and their knowledge of viral infections. Vaccines.

[19] Santangelo O.E., Provenzano S., Firenze A. (2021). Factors influencing flu vaccination in nursing students at Palermo University. J. Prev. Med. Hyg..

[20] Keisala J., Jarva E., Comparcini D., Simonetti V., Cicolini G., Unsworth J., Tomietto M., Mikkonen K. (2025). Factors influencing nurses and nursing students’ attitudes towards vaccinations: A cross-sectional study. Int. J. Nurs. Stud..

[21] Census and Statistics Department, The Government of the Hong Kong Special Administrative Region Mid-Year Population for 2025 [14 August 2025]. https://www.censtatd.gov.hk/en/press_release_detail.html?id=5614.

[22] Centre for Health Protection of the Hong Kong Special Administrative Region (2025). Seasonal Influenza Vaccination. https://www.chp.gov.hk/en/features/107880.html.

[23] Centre for Health Protection of the Hong Kong Special Administrative Region (2025). Other Eligible Groups for SIV. https://www.chp.gov.hk/en/features/108125.html.

[24] Hong Kong Special Administrative Region Press Releases. 2024/25 Seasonal Influenza Vaccination Programmes to Start on September 26. https://www.info.gov.hk/gia/general/202409/19/P2024091900520.htm.

[25] University Medical Service Office, The Chinese University of Hong Kong Influenza Vaccination Service 2024–2025. https://www.umso.cuhk.edu.hk/university-medical-service-office-umso-influenza-vaccination-service-2024-2025/#.

[26] Promotions: Flu Vaccine Limited Offer. https://gleneagles.hk/promotions/flu-vaccine-limited-offer-gleneagles-hospital-hong-kong.

[27] Centre for Health Protection of the Hong Kong Special Administrative Region (2025). Vaccination Schemes—Healthcare Workers. https://www.chp.gov.hk/en/features/18886.html.

[28] Lau L.H.W., Lee S.S., Wong N.S. (2020). The continuum of influenza vaccine hesitancy among nursing professionals in Hong Kong. Vaccine.

[29] Wong N.S., Lee S., Lee S.S. (2018). Differing pattern of influenza vaccination uptake in nurses between clinical and long term care facilities setting, 2014–2018. Int. J. Infect. Dis..

[30] Wang K., Wong E.L.Y., Ho K.F., Cheung A.W.L., Chan E.Y.Y., Yeoh E.K., Wong S.Y.S. (2020). Intention of nurses to accept coronavirus disease 2019 vaccination and change of intention to accept seasonal influenza vaccination during the coronavirus disease 2019 pandemic: A cross-sectional survey. Vaccine.

[31] Cheung K., Ho S.M.S., Lam W. (2017). Factors affecting the willingness of nursing students to receive annual seasonal influenza vaccination: A large-scale cross-sectional study. Vaccine.

[32] Wang X., Cheng Z. (2020). Cross-Sectional Studies: Strengths, Weaknesses, and Recommendations. Chest.

[33] Peduzzi P., Concato J., Kemper E., Holford T.R., Feinstein A.R. (1996). A Simulation Study of the Number of Events per Variable in Logistic Regression Analysis. J. Clin. Epidemiol..

[34] Silva S.B., de Oliveira Souza F., de Sousa Pinho P., Santos D.V. (2023). Health belief model in studies of influenza vaccination among health care workers. Rev. Bras. Med. Trab..

[35] Corace K.M., Srigley J.A., Hargadon D.P., Yu D., MacDonald T.K., Fabrigar L.R., Garber G.E. (2016). Using behavior change frameworks to improve healthcare worker influenza vaccination rates: A systematic review. Vaccine.

[36] Nursing Council of Hong Kong (2025). A Reference Guide to the Syllabus of Subjects and Requirements for the Preparation of a Registered Nurse (General) in the Hong Kong Special Administrative Region. March 2025.

[37] Waghmare P.H., Siracuse M.V., Ohri L.K., Bramble J.D. (2023). A survey of university students on attitudes, behaviors, and intentions toward influenza vaccination. J. Am. Coll. Health.

[38] Bertrand S.F., Kok G., Mafi A., Ruiter R.A., Ten Hoor G.A. (2025). Interventions targeting healthcare worker influenza vaccination: A systematic review. Hum. vaccin. Immunother..

[39] Martínez-Serrano A., Pulido-Fuentes M., Notario-Pacheco B., Palmar-Santos A.M., Cobo-Cuenca A.I., Díez-Fernández A. (2025). Immunity Awareness—Strategies to Improve the Degree of Acceptance of Vaccines: A Systematic Review. Vaccines.

[40] Nyandoro M.G., Kelly D.A., Macey D.J., Mak D.B. (2016). Student-centered interventions the key to student health care worker influenza vaccination. Infect. Dis. Health.

[41] Lam K.K., Hung S.Y.M. (2013). Perceptions of emergency nurses during the human swine influenza outbreak: A qualitative study. Int. Emerg. Nurs..

[42] World Health Organization Post COVID-19 Condition (Long COVID). https://www.who.int/news-room/fact-sheets/detail/post-covid-19-condition-(long-covid).

[43] Hong Kong Special Administrative Region Long COVID. https://www.coronavirus.gov.hk/eng/longcovid.html.

[44] Hung M. (2022). Impact of COVID-19 Pandemic on Hong Kong General Population’s Mental Health. Hong Kong J. Ment. Health.

[45] Xiong J., Lipsitz O., Nasri F., Lui L.M., Gill H., Phan L., Li D.C., Iacobucci M., Ho C.M.R., Majeed A. (2020). Impact of COVID-19 pandemic on mental health in the general population: A systematic review. J. Affect. Disord..

[46] Hung M.S.Y., Chan L.C.K., Liu S.P.S. (2022). The Health Impacts and Life Challenges Caused by the COVID-19 Pandemic on Hong Kong Chinese Women. Int. J. Environ. Res. Public Health.

[47] Batra K., Singh T.P., Sharma M., Batra R., Schvaneveldt N. (2020). Investigating the psychological impact of COVID-19 among healthcare workers: A meta-analysis. Int. J. Environ. Res. Public Health.

[48] Hung M.S.Y., Ng W.W.M., Choi E.K.Y. (2022). The Impacts of the COVID-19 Pandemic on Hong Kong Nursing Students’ Mental Health and Quality of Life. Int. J. Environ. Res. Public Health.

[49] Hall C.M., Cotton A., Webster A., Bushell M., Northam H.L. (2025). Seasonal Influenza Vaccination Uptake Among Australian Healthcare Professionals: An Archetype for Success. Vaccines.

[50] Buonomo E., Di Giovanni D., Piunno G., Moramarco S., D’Elpidio G., Vellone E., Gjini E., Carestia M., Ferrari C., Coppeta L. (2025). Vaccine Attitudes, Knowledge, and Confidence Among Nursing, Pediatric Nursing, and Midwifery Undergraduate Students in Italy. Vaccines.

[51] Wright K.B. (2005). Researching Internet-based populations: Advantages and disadvantages of online survey research, online questionnaire authoring software packages, and web survey services. JCMC.

[52] Durmaz A., Dursun İ., Kabadayi E.T. (2020). Mitigating the effects of social desirability bias in self-report surveys: Classical and new techniques. Applied Social Science Approaches to Mixed Methods Research.

